# A corpus-based study of the translation of modal verbs in the three versions of *Shih chi*

**DOI:** 10.3389/fpsyg.2022.1071071

**Published:** 2023-01-06

**Authors:** Lei Yang, Yue Zhang, Manfu Duan

**Affiliations:** ^1^School of Liberal Arts, Shinawatra University, Bangkok, Thailand; ^2^Foreign Languages College, Inner Mongolia University, Hohhot, Inner Mongolia, China

**Keywords:** corpus-based, *Shih chi*, translation, modal verbs, modality

## Abstract

Modality plays an important part in verbal communication, as it can reveal the speaker’s views and perspectives. The modal verb, as an essential component of the modality system, has been a hot research topic and a challenge in academic circles in recent years. Analyzing the modal verbs in a text may indicate the context produced by the translator in the translation and the identity difference between the translator and the reader. In this study, three representative English translations of *Shih chi* are selected for comparative analysis with the original text. With the help of corpus techniques, the paper investigates the differences in the use of modal verbs by various translators, the distribution of quantitative changes and their influence on translations, as well as the historical and cultural backgrounds and identities of the translators that led to these differences. It is found that owing to the different professional identities of the translators and historical and cultural contexts in which they live, the choice of modal verbs and the shifts of modality values in the three translations are distinct, leading to different translation styles.

## 1. Introduction

Modal verbs are a fundamental aspect of the language system, representing the subjectivity of the speaker’s attitude, ideas, perception, compulsion, and desire to comply with his requests. Modality can be traced back to the study of philosophy in ancient Greece, yet despite its long history, not all concerns about modal verbs have been conclusively settled. The complexity of modality itself has always made this study difficult, for it is rarely regular and predictable. In recent years, the study of modal verbs has drawn wide attention; the perception of modal verbs also increases in tandem with the development of relevant studies. Existing studies on modal verbs incorporate a variety of viewpoints, including English translation studies and semantic comparisons.

Though these studies are diverse in research perspectives on modal verbs, their discussions are generally vague, and few of them focus on the particular usage of modal verbs in literary works. *Shih chi*, written by Sima Qian, a great historian of the Western Han Dynasty in China, is China’s first comprehensive history presented in a series of biographies. *Shih chi* records more than 3,000 years of history, from the reign of the mythical Yellow Emperor in ancient times to the fourth year of Emperor Wu of the Han Dynasty. It is a comprehensive and exhaustive book that has significantly impacted the composition of later historical writings. This literary style was eventually adopted by the official dynastic history of China. Therefore, the usage of modal verbs in this work makes this genre a good option for a comprehensive study of modal verbs. As the translation of modal verbs differs across texts, the study of modal verbs in *Shih chi* can enrich the relevant studies to a certain extent. Meanwhile, the emergence and development of the corpus have not only enriched the research methods of modal verbs, but also given a strong impetus to the interest of the research. Therefore, with the three representative English translations of *Shih chi* as the corpora, this study mainly explores the differences and their reasons in the usage of modal verbs among the three translators.

## 2. Modal verbs

### 2.1. Modality types

*Modality* is the speaker’s cognitive, affective, and volitional attitude toward a state that is between “yes” and “no” and expresses the author’s or speaker’s evaluation and attitude (Fowler et al., 1979; Asher, 1995). Halliday (2004) argues that the function of modality is to recognize the uncertainty between “yes” and “no.” The study of modality is an essential component of systemic functional linguistics. Modality may be communicated in a variety of ways, including metaphors, tones, particles, modal verbs, and connected morphemes (Yang, 2006). When the speaker’s attitudes and opinions are represented not by a modal component in a subordinate phrase, but by an independent main clause, the metaphor of modality is utilized.

Halliday (2004) believes that in the congruent form, modality is an addition to the statement, not the proposition itself. Thus, he believes that the mood and the argument or suggestion should be represented in two phrases in a metaphor. Many clauses may be employed to represent the metaphor of modality, such as: “I think” or “I believe.” For example, in the sentence, “I think he will take responsibility for all his mistakes,” “I think” is a modal metaphor clause, not a projecting clause, but a modal adjunct, while the clause, “he will take responsibility for all his mistakes,” is the proposition of the sentence.

Tone of modality involves the speaker’s attitudes and opinions about the position of the expressed event in the possible world. Lyons (1977) argues that tone is a grammatical representation of modality, and Bybee, and S, Fleischman. (1995) hold that the tone refers to the formal grammaticalized category of the verb, which has a modal function and can be used to convey mood. Although there are minor distinctions between tone and mood, this paper does not elaborate on tone. Modal verbs are the most prevalent method for expressing mood among the numerous options available. Modal verbs, also known as *modal auxiliaries*, communicate the speaker’s perspective or subjective view of an action or condition (Zhang, 1986). The prevalence of modal verbs in a variety of genres, as well as their semantic richness and complexity, makes modal verbs a key focus and challenge in the study of English translation (Ma and Li, 2007). Biber et al. (1999) categorized modal verbs in English as core modal, marginal modal, and semi-modal verbs. The core modal verbs include *can*, *could*, *may*, *might*, *must*, *shall*, *should*, *will*, and *would*. The marginal modal verbs include *dare*, *need*, *ought to*, and *used to*. Due to the limitation of space, this paper only makes an in-depth study of some frequently used modal verbs in the English translations of *Shih chi*.

### 2.2. Modality values

The modality of a sentence represents the speaker’s or writer’s cognitive, emotive, and affective attitudes and judgments of the situation (Wei, 2005). People should set mood and tone in communication with attention and sensitivity to ensure that the conversation flows smoothly and that particular communicative goals are achieved. Through the modality values conveyed by modal forms of expression, the speaker can modify the pragmatic direction of the conversation. In addition, analyzing the modality values used by authors in written communication and literary works can identify the social distance and power relations between the speaker and the listener, infer the attitude of the discourse toward events and characters, and help to better explain the interpersonal relations in fictional discourse. Thus, the correct and suitable selection of mood and tone within a discourse can help to narrow the gap between interlocutors and foster a positive self-image throughout the communication process.

Another pair of terms closely related to modality values in sociolinguistics are the *power factor* and the *equivalence relation*, created by social psychologists Roger Brown and Albert Gilman. In terms of position, income, and identity, the power factor refers to the dominant side. This side has a tendency to dominate the discourse, has a more forceful tone of voice, and often uses high modality values. This compels the weaker side to follow and to only contribute to the dialog of the stronger side in a more subdued manner.

Equivalence relation refers to the fact that both sides in the dialog have comparable social rank, seniority, and money, and see each other as partners. Therefore, they do not increase or reduce the modality values in the dialog, but maintain balance when interacting with each other. Interpersonal communication can be broadly divided into the power factor and the equivalence relation; however, in real life and in literary works, the relationship among actual social characters or fictional characters is complex and diverse, and most of the time, it cannot be generalized due to differences in reality.

Halliday (1994) categorized modality as having high, medium, and low values. Modal verbs with high values are *must*, *ought to*, *need*, and *have/has/had to*; the modal verbs with medium values are *will*, *would*, *shall*, and *should*; and the modal verbs with low values are *may*, *might*, *can*, and *could*. In general, the higher the modality value, the more imperative, less deliberative, and more impolite it is; conversely, the lower the modality value, the less imperative, more deliberative, and more polite it is. For example: (1) “You *must* go now” vs. (2) “You *may* go now.” In the two sentences above, the modality value of “*must*” is significantly higher than that of “*may*,” so the first sentence contains a kind of imperative tone and there is no room for negotiation; in the second sentence, the listener is free to make his or her own decision, and the tone is polite and respectful.

In the process of translation, there is a common phenomenon known as the *translation shift*, which can bring small inconsistencies from the source language to the target language (Munday, 2001). The translation of modal verbs in literary works also demonstrates shifts of modality values. The shifts of modality values can be further categorized as *intensive shift*, *weakening shift*, and *equivalence shift* (Li, 2018). Intensive shift means that the translator translates modal verbs with low values into modal verbs with high values; weakening shift means that the translator translates modal verbs with high values into modal verbs with low values (Zhao et al., 2020). Equivalence shift means that the translated modal verbs have the same values as those in the original text (the translator does not change the modality values in the translation process). There are many examples of modality value shifts, which vary considerably across various text types, rendering it necessary for researchers to perform a more specific analysis of these differences.

## 3. Research design

### 3.1. Selection of the corpora

The corpora used in this study are drawn from three representative English translations: *Records of the Grand Historian of China* by the American sinologist Burton Waston, *The Grand Scribe’s Records* by the American sinologist William H. Nienhauser, and *Selections from Records of the Historian* by the Chinese translators Yang Hsien-yi and his wife Gladys Yang.

*Shih chi* has been translated into different languages, which has greatly promoted the global dissemination of Chinese classical records and culture. In the process of translation, the translator must take into account not only the source language and its cultural characteristics, but also the target language and its cultural factors, focusing on the reader’s acceptance and attaining the desired impact (Wei, 2020). Watson translated *Shih chi* for the first time on a broad scale, including an English version, widely celebrated for its huge impact on the study of *Shih chi* in the West (Li, 2006a). UNESCO has included this translation as one of the representative works in the Chinese Series (Li, 2006b). He translated 66 of *Shih chi*’s most literary chapters, of which 57 were translated in their entirety, and nine were in an abridged form (Li, 2015a). Instead of adhering to the original sequence of *Shih chi* in terms of the primary chronicles, chronology, books, sages, and biographies, Watson employed his own creative approach to the arrangement. He divided the entire translation into three parts: the creation of the Han Dynasty, the second phase of the Han Dynasty, and the reign of Emperor Wu. This is because *Shih chi* involves a large number of names, places, and events that are difficult for Western readers to read and comprehend. Therefore, Watson restructured the order of the chapters in the original text, so that the whole translation would be more consistent with the habits of Western readers (Li, 2006a). This is one of the distinguishing characteristics between Watson’s translation and other translations. In addition, his translation relied on the work of the renowned Japanese sinologist Takigawa’s *Shiki kaichū kōshō*, and also linked *Shih chi* with the classics of Western history (Li, 2020). Thus, Watson attaches great importance to the readability of the translation, a key factor in the selection of Watson’s translation as the corpus for this study.

Nienhauser’s translation is a complete version achieved through teamwork. Different from Watson’s translation, Nienhauser’s translation adheres to the original order of *Shih chi*; his textual research on historical facts is quite thorough, and the cultural background and source of the original text are noted at the back of the translation. In addition, his translation is rigorous and boasts high reference value in academic circles, making it a better option for Western researchers when studying China’s history and culture. Nienhauser’s translation has also been lauded by overseas sinologists, including the renowned sinologist Grant Hardy (1996), who comments: “The significance of Nienhauser’s contribution is indelible; *Shih chi* is a foundational work of Chinese history, and this translation provides a complete annotated version of *Shih chi for* students and researchers. Therefore, no one can deny that Nienhauser’s translation is a remarkable achievement and will surely be handed down forever. Moreover, this translation is a model of collaborative translation by an international team.”

Nearly contemporaneous with Watson, famous Chinese translators Yang Hsien-yi and his wife also began their translation of *Shih chi*, selecting 31 classical chapters. Yang has included as much information as possible in the translation, such as the context of the original text, offering a detailed explanation of the background of the original text and also paying attention to the sentiments of the readers. Yang’s translation has earned great appreciation in Chinese academic circles, and it is said to be the first large-scale and generally recognized English translation published in the Chinese mainland (Gao, 2017).

The selection of these three translations as corpora is based on the fact that they are all masterworks with unique qualities and worthy to be studied, although coming from various periods and contexts. *Shih chi*’s original Chinese text is so lengthy that it would be difficult to perform a comparative analysis of all of its chapters. Therefore, in the following comparison analysis, 12 chapters with an average length of 100,000 words are chosen. The selected parts include: *The First Emperor of Chin, Hsiang Yu*, *Tien Tan*, *Han Hsin—Marquis of Huaiyin*, *Chi Pu and Luan Pu*, *Liu Pi—Prince of Wu*, *The Princes of Huainan and Hengshan*, *Chang Shih-Chih and Feng Tang*, *The Marquises of Weichi and Wuan, Li Kuang, The Gallant Citizens,* and *The Harsh Officials*. The average number of words in the three translations of the selected parts is around 100,000, while the average number of modal verbs is 908, with an average proportion of 0.951%. The number of Chinese characters in the original text is about 67,782, while the number of modal verbs is 636, with an average proportion of 0.938%. Moreover, the study only analyzes the textual parts of the translations; the paratextual parts are not included in the corpus. Therefore, the corpora of this study are made by selecting the modal verbs used in the relevant chapters of the translation texts by the three translators, which can reflect the characteristics of the three translated texts, making this study representative and reliable.

### 3.2. Research questions

This paper investigates the choice of modal verbs and the shifts of modality values in the three translations and analyzes the differences in and the reasons for the usage of modal verbs among the three translators. This paper aims to answer the following questions:

1. What are the similarities and differences in the choice of modal verbs among the three translators?
2. What effect do the shifts of modality values have on the three translations?
3. What are the motivations for the differences in the use of modal verbs among the three translators?

### 3.3. Research methods and procedures

Firstly, after extensive proofreading, translated texts and their corresponding source texts were loaded into AntConc in order to create statistics of types and tokens, as well as the frequency of modal verbs. Excel is used to categorize, select, and calculate the statistical results. Secondly, the distribution and proportion of the different values of modal verbs in the original and the translated texts were counted, and the shifts of the modality values from the original text to the translated text were calculated. After collecting the data above, the three translations were compared and analyzed. Finally, the differences of modal verbs used by the three translators were summarized, and the reasons behind the differences in the translators’ styles were further analyzed.

In this study, 13 frequently used English modal verbs were selected, and the proportions of modality values in the three translations were counted according to classification of modality values of Halliday (1994) (Table 1). It is worth noting that the modality values of the affirmative and negative forms of modal verbs in English are identical (Gu, 2021). Due to the limitation of space, only the affirmative forms of modal verbs are analyzed in the subsequent section.

**Table 1:** Halliday’s classification of modality values in English.

Modality value	Modal verbs
High	Must, ought to, need, have/has/had to
Medium	Will, would, shall, should
Low	Can, could, may, might, dare

This study provides a summary of ancient Chinese modal verbs based upon an analysis of the modal verbs in the original text that correlate with the translation. By checking the ancient Chinese lexicon, a number of modal verbs with complicated, unusual, or negative forms or multiple meanings were deleted. For words with less than 150 entries in the AntConc statistics, non-modal usages were deleted by carefully reading the text; for words with more than 150 entries, irrelevant data were excluded by the use of index-related collocations. Based on the steps above, 13 frequently used Chinese modal verbs in the original text were summarized, and these modal verbs were assigned with reference to the Chinese modality value table (Table 2) constructed by Xu (2018) on the basis of Li (2004) and Xu (2007).

**Table 2:** Corresponding modality values in the original text.

Modality value	Modal verbs
High	必bi, 得dei, 须xu
Medium	宜yi, 当dang, 欲yu, 宁ning, 肯ken
Low	可ke, 足zu, 能neng, 敢gan, 恐kong

### 3.4. Result analysis

#### 3.4.1. Overall characteristics of modal verbs

Modal verbs can reflect the diverse perceptions and attitudes of different translators toward the original text. After statistical analysis, this study provides a summary of the frequent modal verbs, their frequency in the original text, and their respective translations (Table 3).

**Table 3:** Common modal verbs in the original text corresponding to the three translations.

Modal verbs	欲yu(198), 必bi(88), 可ke(80), 得dei(53), 当dang(49), 能neng(48), 足zu(38), 敢gan(32), 恐kong(25), 宁ning(12), 宜yi(8), 须xu(3), 肯ken(2)
Total	636
Token	67,782
Proportion	0.938%

According to the overall characteristics of the modal verbs in the original text of *Shih chi* corresponding to the selected translation, the overall usage ratio of modal verbs is relatively low. High-frequency modal verbs include “欲” “必” “可” and low-frequency modal verbs include “宜” “须” “肯.”

In terms of the overall characteristics of the modal verbs in the three translations (Table 4), the high-frequency modal verbs in all three translations are *will* and *would*, which are consistent with the original text. The low-frequency modal verbs are *ought to* and *have/has/had to*, and *need* does not even appear in the form of modal verbs in all three translations. The low frequency of *ought to* in the three translations is mainly due to its similarity to *should* in meaning, and both words mean “应该” in Chinese, but *should* is used much more frequently than *ought to*. In contrast, the former is more context-restrictive than the latter, which is also more prevalent. The frequency of *should* and *ought to* indicates that the three translators are consistent in their choice of modal verbs when expressing the modality meaning of “应该.”

**Table 4:** Common modal verbs in the three translations.

Modal verbs	Watson’s translation	Nienhauser’s translation	Yang’s translation
Will	252	252	183
Would	289	227	110
Can	85	73	103
Should	90	66	96
Could	105	116	59
May	92	16	35
Shall	47	20	45
Must	47	18	53
Need	0	0	0
Might	24	12	27
Have/has/had to	15	13	13
Dare	54	50	30
Ought to	6	1	1
Total	1,106	864	755
Tokens	111,287	99,933	76,035
Proportion	0.994%	0.865%	0.993%

Judging from the proportion of the use of modal verbs, Nienhauser’s translation accounts for the lowest proportion among the three translations, which is quite different from the original text of *Shih chi*, while the proportion in Watson’s translation is the highest and Yang’s translation is more similar to the original text. Palmer (1990) holds that people’s use of modal verbs is subjective at a semantic level, indicating their views or subjective assumptions. Therefore, a translated text with a lower proportion of modal verbs can reflect the translator’s less subjective intervention in the original text, while one with a higher proportion of modal verbs can reflect the translator’s more intensive interpretation of the original text. Thus, Watson’s translation with the highest proportion of modal verbs may reflect more of the translator’s interpretation and concept of the original text, while Nienhauser’s translation with the lowest proportion of modal verbs represents the authority and seriousness of the translation. The following examples illustrate that Watson’s translation uses more modal verbs than the other two translations.

**Example 1**

**ST:**  朕尊万乘，毋其实，吾欲造千乘之驾，万乘之属，充吾号名。
**Watson’s translation:** Now I want to be provided with 1,000 chariots, to be attended by 10,000, so that I *can* live up to my name and title!
**Nienhauser’s translation:** I want to have an escort of one-thousand chariots and followers in tens of thousands of chariots to substantiate my title.
**Yang Hsien-yi’s translation:** In name, we are the lord of ten thousand chariots, but not in fact. So I want a retinue of a thousand, no, ten thousand, chariots to live up to my title.

**Example 2**

**ST:** 普施明法，经纬天下，永为仪则。大矣哉!
**Watson’s translation:** How great, that throughout the whole universe the will of the sage *should* be heeded and obeyed!
**Nienhauser’s translation:** Great is he! Within the red lands under the celestial dome, everyone adopted and followed his mind.
**Yang Hsien-yi’s translation:** Great is he, indeed! The whole universe obeys his sagacious will.

According to the general characteristics of the use of modal verbs, the average proportion of modal verbs used in the three translations is 0.951%, which is very close to 0.938% of the original text of *Shih chi* (Table 4). Moreover, comparing the use of modal verbs in the three translations by SPSS 26.0, the results of the one-way ANOVA show that the variances of the groups are not significantly different at the 0.05 level of significance, i.e., the variances are consistent (sig = 0.661 > 0.05), meaning that there is no significant difference in the use of modal verbs in the three translations (Table 5). Meanwhile, it also demonstrates that the English translations of *Shih chi* have reproduced the original text to a large extent, without much subjective intervention by the translators.

**Table 5:** The differences in the use of modal verbs in the three translations.

	Sum of squares	df	Mean square	F	Sig.
Between groups	4965.282	2	2482.641	0.419	0.661
With groups	213365.077	36	5926.808		
Total	218330.359	38			

#### 3.4.2. The analysis of modality values

The differences in the choice of modal verbs by the translators can also be reflected in the distribution and shifts of modality values in the translated text. The distribution of modality values in the original text (Table 6) is dominated by the low and medium modality values, which indicate the status difference between the author and the readers of the original text. As a great historian in China, Sima Qian wrote *Shih chi* in order to educate Emperor Wu of Han Dynasty and later emperors on historical facts. The fact that the main target readers of *Shih chi* are emperors also leads the author to use modal verbs with medium or low modality values in order to convey a euphemistic and respectful tone in his composition. However, Sima Qian also uses a small number of modal verbs with high modality values in the original text, showing the author’s firm loyalty to advising the emperor and his sincere devotion to the prosperity of the country.

**Table 6:** The distribution of modality values in the original text corresponding to the three translations.

Modality value	Proportion
High	22.64%
Medium	42.30%
Low	35.06%

The percentages of the total number of high, medium, and low modality values in the three translations are sequentially keyed into the one-way ANOVA in SPSS 26.0. The results are shown in Table 7, where the differences in the use of the modality values in the three translations are notable at the 95% confidence interval, as indicated by the significant value (sig = 0.001 < 0.05).

**Table 7:** The differences in the use of modality values in the three translations.

	Sum of squares	df	Mean square	F	Sig.
Between groups	392288.889	2	196144.444	28.154	0.001
With groups	41800.667	6	6966.778		
Total	434089.556	8			

The distribution of modality values in the three translations (Table 8) also focuses on the medium and low values, which is consistent with the original text. Yang’s translation uses more modal verbs with high modality values, rendering his translation more imperative and less deliberative.

**Table 8:** The distribution of modality values in the three translations.

Modality value	Watson’s translation	Nienhauser’s translation	Yang’s translation
High	6.15%	3.70%	8.87%
Medium	61.30%	65.39%	57.48%
Low	32.55%	30.90%	33.64%

In terms of the shifts of translations’ modality values (Table 9), all three translations show a shift from high to medium modality values. Yang’s translation has the lowest proportion of loss of high modality values and the lowest proportion of shift of low and medium modality values. In addition, the distribution of modality values in Yang’s translation is the closest to that of the original text, reflecting that Yang’s translation is the most faithful to the original text among the three translations. In contrast, Nienhauser’s translation has the highest proportion of loss of high modality values and the highest proportion of shift of low and medium modality values, indicating that Nienhauser may integrate a lot of his own views and understandings to his translation.

**Table 9:** The proportion of modality value shifts in the three translations.

Modality value	Watson’s translation	Nienhauser’s translation	Yang’s translation
High	−16.49%	−18.74%	−13.77%
Medium	+19%	+23.09%	+15.18%
Low	−2.51%	−4.16%	−1.42%

#### 3.4.3. The reconstruction of interpersonal function and tenor

In functional linguistics, the interpersonal function of language refers to the way people use language to express their interaction with others and to participate in situations in which their roles convey the identity, status, and attitude of the participants. In any specific discourse, an interpersonal relationship involves the relationship between the author and the readers. Modal verbs are an important form to reproduce interpersonal meaning and narrative features. Therefore, the study of modal verbs is conducive to further analyzing the interpersonal functions in translation.

In terms of the interpersonal function of the original text, since *Shih chi* is written by Sima Qian to Emperor Wu of the Han Dynasty as well as to later emperors, the relationship between the author and the reader is that between the ruler and his subject, and the author’s status is lower than that of the reader. Therefore, Sima Qian uses a large number of modal verbs with low and medium values in writing *Shih chi*, giving the text a euphemistic tone in order to express his reverence for Emperor Wu of the Han Dynasty.

However, the three translations have a significant loss of high modality values, which may be due to the shift of target readers: the primary readers of the English translation of *Shih chi* are not emperors but English-speaking readers all over the world. The translator is more concerned with restoring the historical facts and conveying the author’s thoughts than with attaining the exhortation’s intended objective. Therefore, the three translators have intentionally reduced the proportion of high modality values in their translations, thus narrowing the distance between them and their readers, increasing the participation of readers, and making their translations better serve target readers. In addition, the three translators have also achieved the purpose of reconstructing the interpersonal function through the use of modal verbs.

The choice of modal verbs and the shifts of modality values in translation can reconstruct the interpersonal relationship and the tenor of the translation to some extent (Xu and Nie, 2021). *Tenor*, also known as “tone of discourse,” refers to the relationship between the participants. In addition, tenor marks the relationship between the two parties, their basic situation, characteristics, status, and their roles in the communication, but the different roles also contribute to varied linguistic styles of the text.

Nienhauser’s translation is intended for the academic readers and provides detailed and valuable historical materials of China for Western researchers, so it must be translated in a rigorous and standardized manner to reflect its value as a reference for academic research. In order to demonstrate the seriousness of the historical text, Nienhauser uses a lower proportion of modal verbs in general. This results in a higher overall modality value of the translation, and a more distant interpersonal relationship between the translator and the target readers, leading to a higher degree of linguistic formality in translation and a reconstruction of an “academic translator-research reader” tenor. In contrast, Watson strives to improve accessibility and readability. His translation is aimed at the common readers, rather than professionals (Li, 2006a). In order to convey the explanatory nature of the original text, Watson generally uses a larger proportion of modal verbs in his translation, presenting an interpretation of historical materials. Watson’s tendency to use modal verbs with low and medium values reduces the overall modality values of the translation, reflects the closer interpersonal relationship with the target readers, makes the language less formal, and reconstructs an “expert translator-general reader” tenor.

## 4. The motivation analysis of translation differences of modal verbs in *Shih chi*

### 4.1. The internal cause: Translators’ identities

Wong and Shen (1999) argue that translating bridges the cultural gap between two worlds and makes communication possible between different linguistic communities. Translators from different professional backgrounds and cultural and historical contexts may have an impact on their translation.

Born in New York, Watson joined the United States Navy during the Second World War and arrived in Japan, where he first became interested in Eastern culture. After retiring from the army, Watson studied Chinese and Japanese literature at Columbia University, where he obtained his doctorate. For more than half a century, Watson devoted himself to English translation of Chinese and Japanese classical literature, and proceeded to translate many great literary works of high quality throughout his life. His work also promoted the spread of *Shih chi* in the West, and went on to complete more ancient and obscure works as an introduction to the charm of Chinese history and culture for a much wider audience. His translation style has always been straightforward and easy for Western readers to understand, using simple language and expressions. As his target readers are well-educated readers in the West, Watson attempts to reflect *Shih chi* as an excellent narrative literature rather than a historical document with significant research value in his selection of materials and formulation of translation strategies (Li, 2006a). Meanwhile, Watson’s translation is straightforward and attractive, based on the principle of readability. At that time, Eastern historical and cultural works were on the margins of the Western book market, and some translators blindly used foreignization strategies, which demonstrated the heterogeneity of Chinese culture and failed to attract the interest of the ordinary Western readers (Zhao, 2010). It can be said that Watson’s translation has made an important contribution to the spread of Chinese classic history and culture. Compared with Nienhauser’s and Yang’s translations, Watson’s translation pays more attention to enlightening the readers, so he uses a larger proportion of modal verbs in general. In the process of translation, he integrates his emotions and opinions into his translation, analyzes history with his own unique perspective, and reduces the barrier between Western readers and obscure China classics, to enable more Western readers to appreciate Chinese history and culture.

Nienhauser, a renowned American sinologist, has been engaged in the study of Chinese literature and devoted himself to the translation of many Chinese classical works for a long time. He has an extensive understanding of Sinology and is proficient in several languages. In addition, he traveled to China, Japan, and France to study and discuss the translation of *Shih chi* in order to ensure its accuracy. After years of studying *Shih chi*, Nienhauser realized that it should be translated into a complete and detailed literary work with historical reference values. Therefore, he assembled an international translation team led by Sinologists to study the full translation of *Shih chi* (Wei, 2021). This innovative translation method allows individual Sinologists to circumvent the lack of language and culture translation, and the team is strong enough to increase the effect of the translation project. Meanwhile, the cooperation of many Sinologists has enabled translation to be optimized to the greatest extent. Due to Nienhauser’s focus on his target readers, he was particularly rigorous in handling academic concerns, which is fully reflected in the translation of *Shih chi*. In order to avoid subjective interference with historical sources and the scientific and referential nature of the translation, Nienhauser generally used a low proportion of modal verbs, thereby conveying the authority and precision of the historical text.

Yang Hsien-yi was born in a wealthy family and his parents paid high attention to his education. Mr. Wei Ruzhou, his enlightened teacher, taught him Chinese traditional writings such as the *Four Books* and *Five Classics*, and he developed a keen interest in Chinese classical literature. Under the influence of his first teacher, he studied many classics at a young age and laid a solid foundation for Chinese studies. As a Chinese translator, Yang Hsien-yi is quite familiar with the names of characters, feudal systems, official positions, and other cultural terms in *Shih chi*, which led to a high degree of acceptance and recognition of the compilation style in *Shih chi*. It is clear that judging from his translation, he strongly preserved the style of the original text, because the style and structure of this work have the characteristics of Chinese culture, which would be lost if they were altered (Gao, 2019). The translator’s sense of national mission and social responsibility makes Yang Hsien-yi eager to undertake the responsibility of spreading China’s native culture. Moreover, as he was a former employee of the China International Communications Group, Yang’s translation is inevitably aimed at publicity and communication (Feng, 2017). Perhaps influenced by his work habits, Yang Hsien-yi himself believes that translation should not be overly creative or rewritten, but should be extremely faithful to the original text, that is, to express the meaning of the original text in another language and to present the meaning of the translated text as close as possible to the original text. Yang Hsien-yi once said, “There are many other elements in literature that constitute some meanings of the original text, and it is impossible to convey these meanings to people of different cultures” (Lin, 1997). In order to promote the outstanding cultural classics of the Chinese nation and the international spread of Chinese culture, Yang Hsien-yi adopts the strategy of foreignization in the English translation of *Shih chi*, still with the utmost regard for the author and fidelity to the original text. Thus, the overall characteristics of the modal verbs in Yang’s translation are also the most similar to the original text of *Shih chi*, which reflects his desire to be faithful to the original text as much as possible.

### 4.2. The external cause: Historical and cultural contexts

Yang Hsien-yi’s translation is deeply influenced by the historical and cultural contexts in which he lives. Supported by China International Communications Group, Yang’s translation has strong political overtones. The Group’s primary responsibility is to encourage newspaper coverage and publicity in other nations; it is the state’s official and specialized international publicity agency, whose mission is to promote political propaganda and ideological dissemination (Li, 2021). The translation works of Yang were made in the 1950s to dispel Western misconceptions about Chinese culture. Therefore, at that time, some Chinese translators translated many historical texts into English for dissemination, so that the West might learn about the New China, and a positive worldwide image of China could be established. Therefore, Yang’s translation came into being under the historical and cultural context of introducing China to the world, spreading Chinese culture, and establishing a positive national image.

Watson’s translation reflects the post-colonial context, which was one aspect of the historical and cultural context of the United States at that time. After the Second World War, Sino-U.S. relations deteriorated. At that time, the United States was a superpower and sought to impose its ideology on Third World countries to enhance its cultural dominance around the globe. As a result of this dominant ideology, translators at that time attached great importance to “fluency” and mainly adopted the strategy of domestication (Man, 2015). This was mostly due to British and American cultural hegemony, reflecting their lack of respect for and understanding of other cultures. Watson’s translation was born in this atmosphere, in which the fluency of the translation cannot be separated from the extensive use of domestication translation strategy. At that time, the translator’s pursuit of fluency and readability might lead to the omission of some culturally-loaded words and obscure cultural customs in the source language or the replacement by elements from Western countries. However, it was in the era when Eastern culture was ignored that Watson’s translation made a significant contribution to introducing Chinese history and culture to the Western world.

Nienhauser’s translation was carried out against the background of deepening international communication. By the 1970s, due to the dramatic transformation in Sino-U.S. relations, more Americans were enthusiastic about Chinese culture, and American sinologists were more engaged in academic research on Chinese history (Li and Xiao, 2016). Since the 1990s, the rapid globalization has brought not only economic and political contacts, but also cultural exchanges. The wave of globalization has fostered cultural exchanges between countries, and created a context of multicultural coexistence. At this time, translation has also been subtly influenced, becoming an effective way for people to learn more about different cultures. During this period, as cultural exchanges between countries became more frequent, Nienhauser’s translation was sponsored by many Chinese and international specialists and organizations (Li, 2015b). In the end, it was published by the prestigious Indiana University Press in the United States and widely disseminated around the world. Therefore, the Nienhauser’s translation originated in this rather open-minded cultural context.

In general, these three translations are products of different historical and cultural contexts, but they have their own merits and values. Together, they have promoted the dissemination and reception of *Shih chi* in the West and made great contributions to the popularization of Chinese history and culture around the world.

## 5. Conclusion

Modality is an essential component of language communication and has always been the focus in pragmatics. The modal verbs are an important part of the modality system, and can convey a great deal of information from different perspectives. This research forms a comprehensive corpus-based analysis of the modal verbs in the three translations of *Shih chi*. First, it is found that Yang Hsien-yi’s translation is the closest to the original text in terms of the overall proportion of modal verbs and that his translation is also the most faithful to the original text of *Shih chi*. Second, the proportion of modal verbs in Nienhauser’s translation is the lowest, reflecting its precision and scientific nature as a historical document. Third, the overall proportion of modal verbs in Watson’s translation is the highest, manifesting the addition of Watson’s own insights to his translation and its enlightening and interpretive orientation.

This study also investigates the motivations behind these differences. Watson translated *Shih chi* during a period when Sino-U.S. relations were tense, and at that time, Anglo-American cultural hegemony made it difficult for Eastern culture to spread worldwide. Moreover, Watson’s translation was somewhat influenced by the dominant ideology and the prevalence of fluency and readability in Western cultural circles. Nienhauser’s translation was completed at a time when Sino-U.S. relations were good and international cultural exchanges were frequent. Therefore, that period was in a relaxed and free cultural environment, which facilitated the dissemination of Nienhauser’s translation to a certain extent. Yang Hsien-yi’s translation was somewhat politically oriented, because China International Communications Group hoped to spread Chinese culture to the Western world by translating a number of historical classics. Therefore, the differences in the use of modal verbs in a work and their influence on translation are closely related to the translators’ personal identities and the historical and cultural contexts in which they live.

In addition, there are still a lot of discussion and research fields of modal verbs to be explored. This study only analyzes the influence and reasons of the differences in the use of modal verbs in the English translation of the historical document *Shih chi*. The role of modal verbs in the translation of various genres should be further studied to analyze their influence on the translator’s style, and whether there is a reflection of interpersonal function and tenor in the translated text.

## Data availability statement

The original contributions presented in the study are included in the article/supplementary material, further inquiries can be directed to the corresponding author.

## Author contributions

All authors listed have made a substantial, direct, and intellectual contribution to the work and approved it for publication.

## Conflict of interest

The authors declare that the research was conducted in the absence of any commercial or financial relationships that could be construed as a potential conflict of interest.

## Publisher’s note

All claims expressed in this article are solely those of the authors and do not necessarily represent those of their affiliated organizations, or those of the publisher, the editors and the reviewers. Any product that may be evaluated in this article, or claim that may be made by its manufacturer, is not guaranteed or endorsed by the publisher.
